# Superior mesenteric aneurysm associated with median arcuate ligament syndrome and a single celiacomesenteric trunk

**DOI:** 10.1016/j.jvscit.2023.101348

**Published:** 2023-10-10

**Authors:** Drew J. Braet, Kian Pourak, Frank M. Davis, Jonathan L. Eliason, Chandu Vemuri

**Affiliations:** a Section of Vascular Surgery, Department of Surgery, University of Michigan, Ann Arbor, MI; b Department of Surgery, University of Michigan, Ann Arbor, MI

**Keywords:** Aneurysm, Celiac artery, Median arcuate ligament syndrome, Superior mesenteric artery

## Abstract

Median arcuate ligament syndrome (MALS) is known to promote arterial collateral circulation development from mesenteric vessel compression and can lead to the development of visceral aneurysms. These aneurysms are often diagnosed at the time of rupture and pose a significant morality risk without appropriate intervention. A celiacomesenteric trunk is a rare anatomic variant in which the celiac artery and superior mesenteric artery share a common origin and has been postulated as a risk factor for developing MALS. In this report, we present a novel case of MALS in a patient with a celiacomesenteric trunk and a superior mesenteric artery aneurysm.

Median arcuate ligament syndrome (MALS) is a rare condition, stemming from compression of the proximal celiac artery (CA) and splanchnic nerves from the median arcuate ligament (MAL).^1^, ^2^, ^3^ The median arcuate ligament (MAL) is composed of the arcuate ligament of the diaphragmatic crura and forms an attachment from the diaphragm to the vertebrae. When the MAL courses across the aorta, the fibers can encircle the CA and compress it and the celiac plexus (CP), resulting in a constellation of symptoms (eg, postprandial abdominal pain and weight loss) known as MALS.^1^, ^2^, ^3^ The presence of a celiacomesenteric trunk (CMT), a rare anatomic variant in which the CA and superior mesenteric artery (SMA) share a common origin, has been postulated as a risk factor for the development of symptoms of MALS.^4^ Visceral artery aneurysms (VAAs) are associated with MALS and are presumed to be secondary to compensatory collateral circulation to circumvent the mesenteric vessel compression, compounding post-stenotic dilatation.^5^, ^6^, ^7^ Post-stenotic dilation has been postulated to have both genetic- and hemodynamic-mediated contributions and is seen in various arterial beds downstream of stenotic and compressive lesions or significant flow disturbances.^8^, ^9^, ^10^, ^11^, ^12^ We present a novel case of MALS in a patient with a CMT and a SMA aneurysm.

## Case report

**Fig 1:**
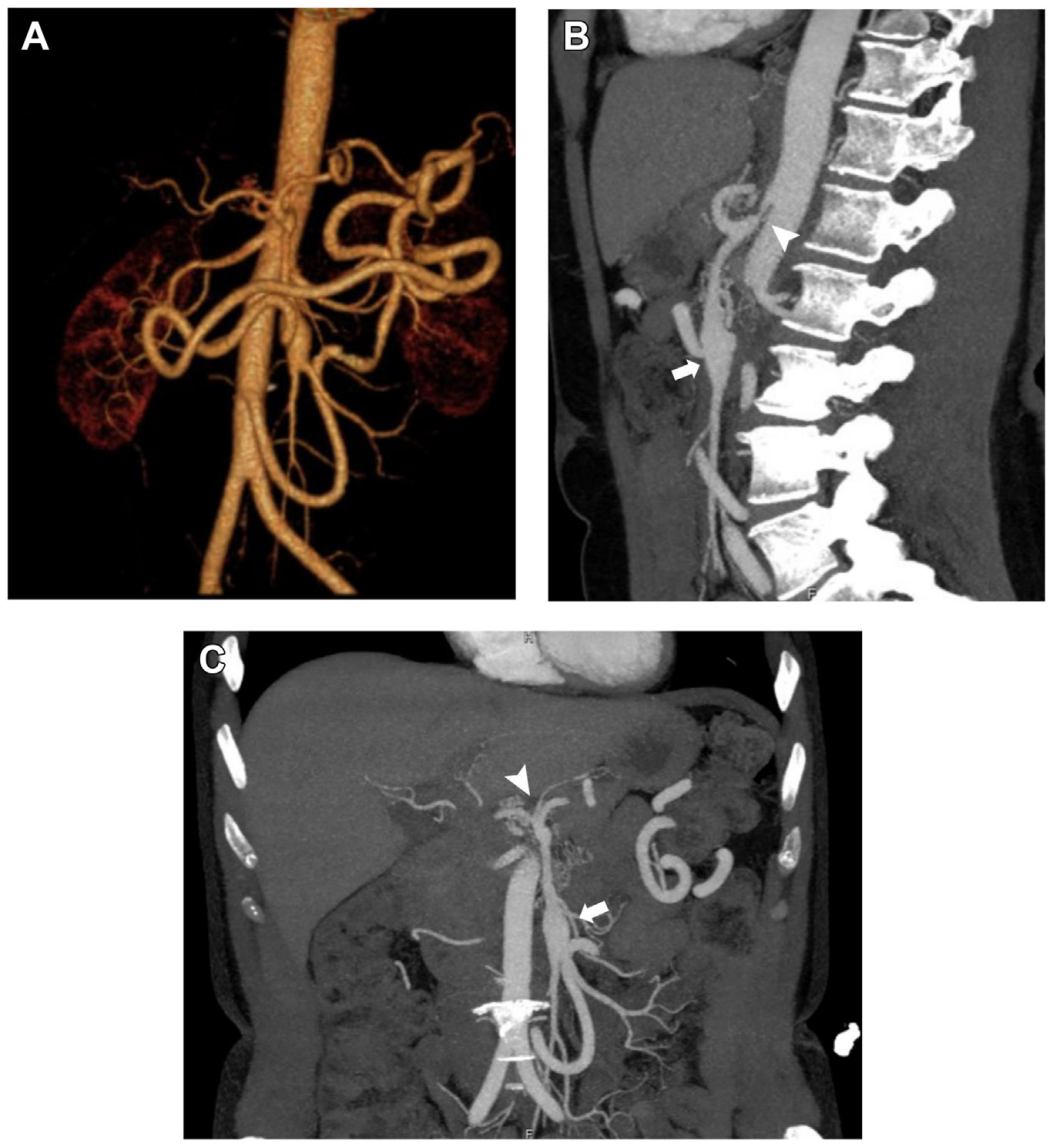
Preoperative computed tomography angiograms. **A,** Three-dimensional reformatted computed tomography angiogram demonstrating a celiacomesenteric trunk (CMT), large inferior mesenteric artery collateral vessel, and a 1.8-cm superior mesenteric artery (SMA) aneurysm. **B,** Sagittal computed tomography angiogram cut depicting origin of the CMT with compression (*white arrowhead*) and SMA aneurysm (*white arrow*). **C,** Coronal computed tomography angiogram cut depicting CMT (*white arrowhead*) and SMA aneurysm (*white arrow*).

**Fig 2:**
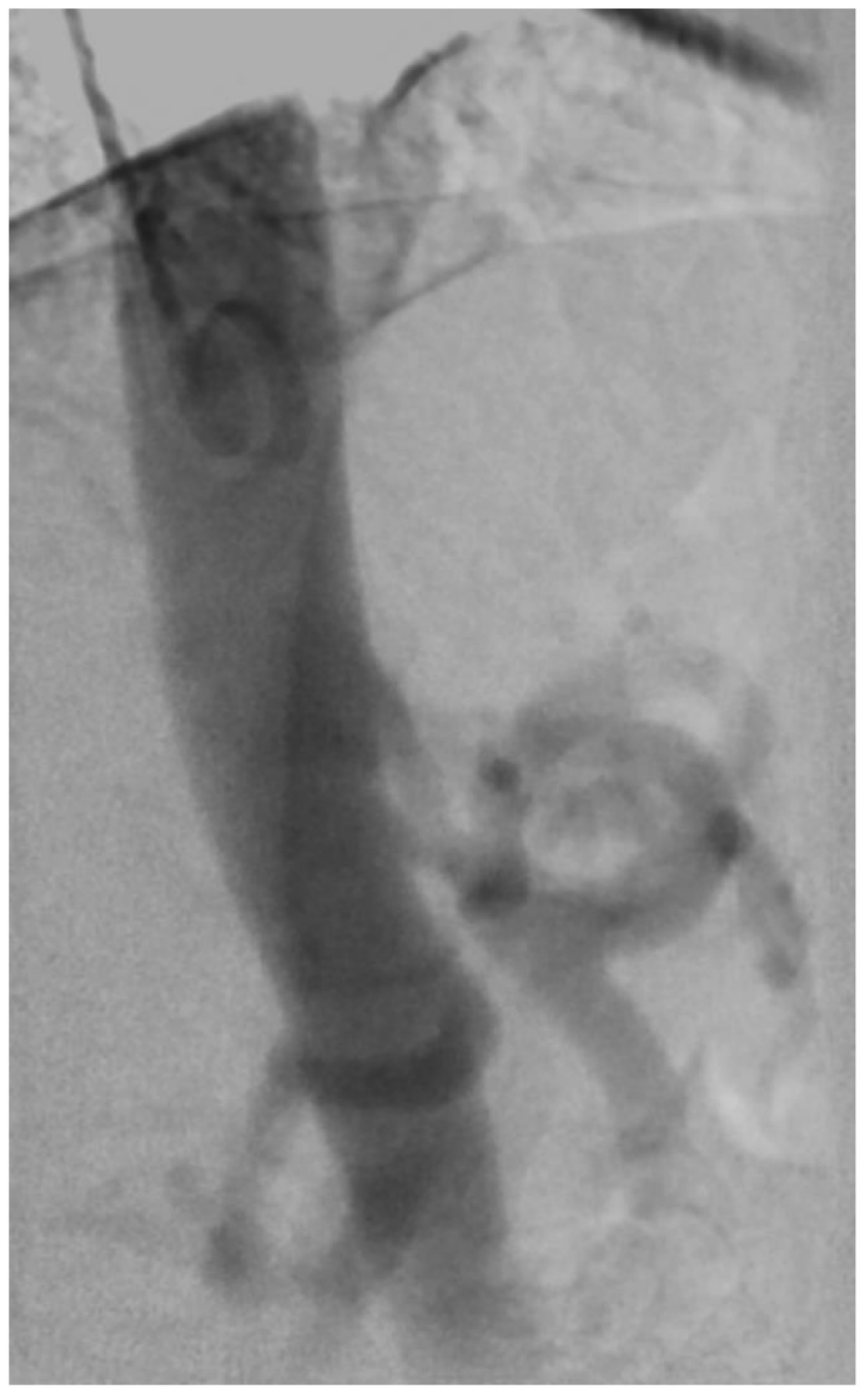
Angiogram demonstrating severe stenosis of the celiacomesenteric trunk (CMT) during end inspiration.

A 50-year-old woman with a history of hypertension, hyperlipidemia, tobacco use, and coronary artery disease was referred for MALS. The patient reported a long history of postprandial abdominal pressure, nausea and vomiting, and unintentional weight loss. Her medications included a proton pump inhibitor, cholesterol medication, and antiplatelet therapy. Examination revealed a soft, nontender, and nondistended abdomen. Workup for a gastrointestinal etiology was negative, including upper endoscopy, right upper quadrant ultrasound, and evaluation for inflammatory bowel disease. Mesenteric duplex ultrasound demonstrated a CA peak systolic velocity of 522 cm/s and a SMA peak systolic velocity 135 cm/s. Dynamic measurements were not obtained. Computed tomography demonstrated a CMT with compression from her MAL (Fig 1), a large inferior mesenteric artery (IMA) collateral vessel, and a 1.8-cm SMA aneurysm (Fig 1). Angiography demonstrated severe stenosis of the CA on the inspiration view and occlusion on the expiration view, with hypertrophy of the IMA and reversal of flow into the SMA (Fig 2). After a CP block, the patient reported relief of her symptoms. Given that her SMA aneurysm was believed to be compensatory from her CMT compression, the decision was made to proceed with MAL release without SMA aneurysm repair, because the SMA could remodel over time after the release.^13^, ^14^, ^15^

A midline laparotomy was made from the xiphoid process down to the umbilicus. The subcutaneous tissues were dissected, and the fascia was entered. On entering the abdomen, the small bowel, large bowel, stomach, liver, and gallbladder were inspected and were unremarkable. The gastrohepatic ligament was divided, and the patient's esophagus was retracted to the left. The left lobe of the liver was taken down by dividing the triangular ligament and was retracted toward the patient's right underneath a fixed retractor. The dissection was continued down to the supraceliac aorta. The pancreas was retracted caudally, and the CMT was identified, which appeared narrowed, with post-stenotic aneurysmal dilation of the SMA. The surrounding diaphragmatic bands were incised, with visible relief of the compression, and the nerves from the CP overlying the CMT were excised. The abdomen was closed in standard fashion.

**Fig 3:**
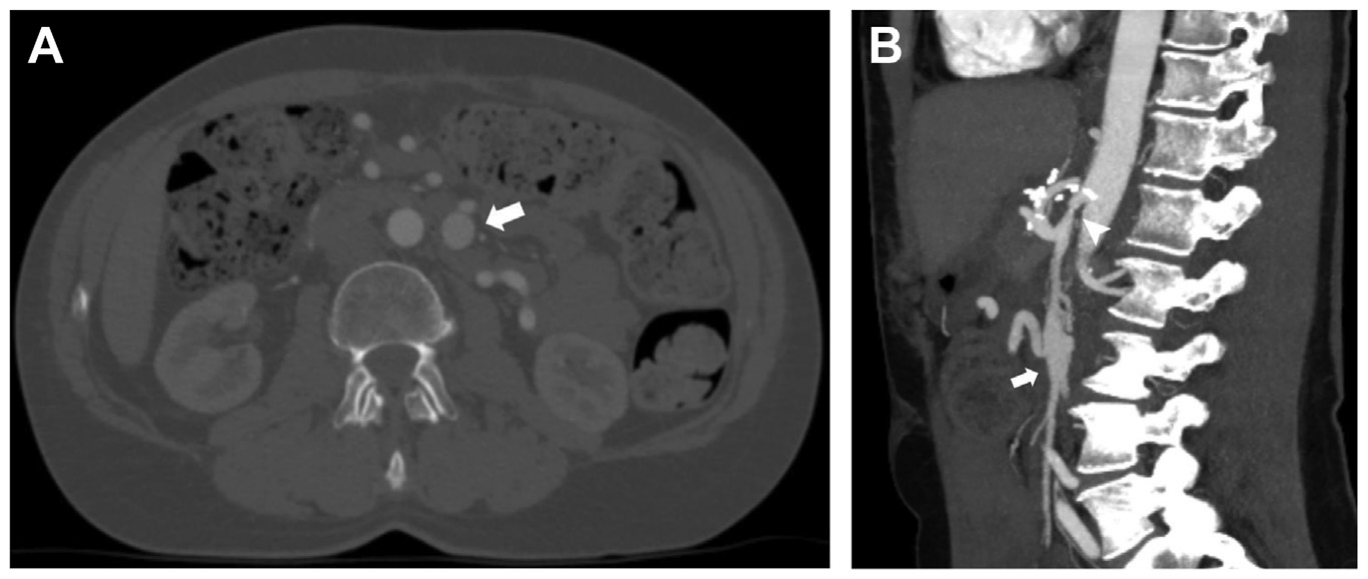
Postoperative computed tomography angiogram obtained at 6-month follow-up. **A,** Axial computed tomography angiogram cut depicting superior mesenteric artery (SMA) aneurysm, 1.8 cm in maximum diameter (*white arrow*). **B,** Sagittal computed tomography angiogram cut demonstrating resolved compression of celiacomesenteric trunk (CMT; *white arrowhead*) and stable SMA aneurysm (*white arrow*).

The patient's postoperative course was uncomplicated. She was discharged on postoperative day 7 able to consume a general diet. At her follow-up visit, the patient reported relief of symptoms and weight gain. Her postoperative computed tomography scan, obtained at the 6-month follow-up, demonstrated resolved CMT compression but a persistent SMA aneurysm, which was stable in size (Fig 3). She has remained asymptomatic postoperatively. She provided written informed consent for the report of her case details and imaging studies.

## Discussion

Mesenteric vessel compression associated with MALS is known to promote arterial collateral circulation development, which can lead to the development of visceral aneurysms.^5^, ^6^, ^7^ Post-stenotic dilation can be seen in vessels that are downstream of critical compression and/or stenoses, such as in the aorta in patients with aortic stenosis, the subclavian artery in patients with thoracic outlet syndrome, and renal arteries in patients with significant atherosclerotic lesions.^8^^,^^12^^,^^16^^,^^17^ Visceral aneurysm formation in patients with MALS has been postulated to be a compensatory mechanism from increased blood flow in the collateral arteries from the SMA to the CA. Up to 46% of patients with MALS develop collateral circulation, and 24% have splanchnic artery aneurysms.^5^^,^^7^ VAAs in patients with MALS are often diagnosed at the time of rupture and can pose a significant mortality risk.^18^^,^^19^ The largest case series of VAAs in patients with MALS demonstrated that inferior pancreaticoduodenal aneurysms were the most common (87.1%), with other VAAs being more rare.^20^ Although SMA dissection associated with MALS has been reported, to the best of our knowledge, no studies have reported SMA aneurysms in patients with MALS.^21^^,^^22^

The presence of a CMT has been hypothesized as a risk factor for the development of symptoms in patients with MALS, because compression of both the CA and the SMA impairs perfusion to a larger area and, thus, is more likely to provoke symptoms consistent with mesenteric ischemia and CP compression.^4^ In patients with MALS, an important collateral pathway between the CA and SMA is through the pancreaticoduodenal and dorsal pancreatic arteries, which are also the most common locations for aneurysms in MALS patients.^5^^,^^20^ It is hypothesized that these arteries, specifically, are affected because they represent a collateral pathway and thus are exposed to higher flow when CA compression is present.^5^^,^^20^ In the case of a CMT, the primary collateral pathway would not be in the pancreaticoduodenal arteries because both the CA and the SMA share a common origin but, rather, would be from the SMA and IMA, thus contributing to hypertrophied collateralization of the SMA and possible aneurysm formation.^9^^,^^10^ This report provides a novel example of a patient with MALS and a CMT and highlights the adaptive vascular response and aneurysm development that can be associated with MALS and the presence of a CMT.

VAAs are rare and constitute only 5% of intra-abdominal aneurysms, with 3.5% to 8% of those representing SMA aneurysms.^23^, ^24^, ^25^ Up to 25% of VAAs present with rupture, which is associated with high rates of mortality.^26^^,^^27^ SMA aneurysms are especially morbid, with rupture rates of 30% to 50% and mortality as high as 90%.^28^^,^^29^ Current guidelines from the Society for Vascular Surgery recommend surgical repair of all SMA aneurysms regardless of size, with an endovascular approach preferred if anatomically suitable.^23^ SMA aneurysms are typically secondary to atherosclerotic, infectious, or inflammatory causes with a more aggressive natural history. However, in the present patient, the SMA aneurysm was thought to have resulted from the abnormal flow due to collateral pathways and thus was not believed to possess risks similar to those for de novo SMA aneurysms. Although the natural history of post-stenotic dilatations and aneurysms is not robustly defined, it is thought that if the dilatation is small, removal of the stenosis could cause reversal of the post-stenotic dilatation.^9^ In contrast, large dilatations can exhibit permanent aneurysmal changes and could warrant repair.^9^ Moreover, guidelines support conservative treatment of patients with post-stenotic dilation of the CA secondary to compression from MALS because of the different pathologic process causing such dilation.^23^ These guidelines also state that these patients require individualized decision-making and should be treated conservatively unless they become symptomatic or are found to have true aneurysmal degeneration.^23^ Although open repair of VAAs has a low morbidity (approximately 1%-2%), complications are common and occur in 10% to 13% of patients.^30^^,^^31^ Thus, the decision was made to proceed with MAL release without SMA aneurysm repair. On follow-up imaging, her SMA aneurysm was stable in size. However, we could not exclude other causes of de novo aneurysm formation and, thus, must continue regular surveillance of her SMA aneurysm. This case further supports that compensatory visceral aneurysms in MALS might be less ominous, although long-term follow-up is still warranted for our patient.

The data on open vs laparoscopic MALS release is heterogenous. A meta-analysis comparing outcomes between open and laparoscopic treatment of patients with MALS demonstrated no difference in recurrent symptoms between the two methods.^32^ Patients undergoing laparoscopic release had a higher percentage of intraoperative complications but a lower incidence of postoperative complications.^32^ Jimenez et al^32^ concluded that both laparoscopic and open ligament release can provide sustained symptom relief for most patients diagnosed with MALS. Other investigators suggest that laparoscopic management of MALS has lower rates of perioperative complications and shorter hospitalization, supporting the laparoscopic technique as a safe option for treating MALS patients.^33^^,^^34^ Despite an increase in the number of laparoscopic repairs, open MALS release remains the most common treatment modality.^32^, ^33^, ^34^ At our institution, we prefer an open approach for MAL release. Patients are counseled on both laparoscopic and robotic options and are referred to the appropriate providers if they prefer a different management option.

## Conclusions

In this report, we present a novel case of MALS in a patient with a CMT and SMA aneurysm. To the best of our knowledge, this is the first report of an SMA aneurysm in a MALS patient, which is a much less common site of aneurysm presentation with this pathology. Additionally, this case further supports the role of a CMT in MALS patients and highlights the different compensatory pathways that can become aneurysmal in these patients. In the present case, the patient clinically improved and her SMA aneurysm has been safely monitored without progression. However, continued clinical and imaging surveillance are required for our patient. Further studies are warranted to determine the role of SMA aneurysm repair in these patients.

## Disclosures

None.
